# Single intranasal immunization with chimpanzee adenovirus-based vaccine induces sustained and protective immunity against MERS-CoV infection

**DOI:** 10.1080/22221751.2019.1620083

**Published:** 2019-05-25

**Authors:** Wenxu Jia, Rudragouda Channappanavar, Chao Zhang, Mingxi Li, Haixia Zhou, Shuyuan Zhang, Panpan Zhou, Jiuyang Xu, Sisi Shan, Xuanling Shi, Xinquan Wang, Jincun Zhao, Dongming Zhou, Stanley Perlman, Linqi Zhang

**Affiliations:** a Comprehensive AIDS Research Center, Collaborative Innovation Center for Diagnosis and Treatment of Infectious Diseases, Beijing Advanced Innovation Center for Structural Biology, Department of Basic Medical Sciences, School of Medicine, Tsinghua University, Beijing, People’s Republic of China; b Department of Microbiology and Immunology, The University of Iowa, Iowa City, IA, USA; c Department of Acute and Tertiary Care, and the Institute for the Study of Host–Pathogen Systems, University of Tennessee Health Science Center, Memphis, TN, USA; d School of Medicine and Life Sciences, Nanjing University of Chinese Medicine, Nanjing, People’s Republic of China; e Key Laboratory of Molecular Virology & Immunology, Vaccine Research Center, Institut Pasteur of Shanghai, Chinese Academy of Sciences, Shanghai, People’s Republic of China; f The Ministry of Education Key Laboratory of Protein Science, Beijing Advanced Innovation Center for Structural Biology, Collaborative Innovation Center for Biotherapy, School of Life Sciences, Tsinghua University, Beijing, People’s Republic of China; g State Key Laboratory of Respiratory Disease, Guangzhou Institute of Respiratory Health, The First Affiliated Hospital of Guangzhou Medical University, Guangzhou, People’s Republic of China

**Keywords:** MERS-CoV vaccine, chimpanzee adenoviral vector, receptor binding domain (RBD), intranasal immunization, monoclonal antibody

## Abstract

The recently identified Middle East Respiratory Syndrome Coronavirus (MERS-CoV) causes severe and fatal acute respiratory illness in humans. However, no approved prophylactic and therapeutic interventions are currently available. The MERS-CoV envelope spike protein serves as a crucial target for neutralizing antibodies and vaccine development, as it plays a critical role in mediating viral entry through interactions with the cellular receptor, dipeptidyl peptidase 4 (DPP4). Here, we constructed a recombinant rare serotype of the chimpanzee adenovirus 68 (AdC68) that expresses full-length MERS-CoV S protein (AdC68-S). Single intranasal immunization with AdC68-S induced robust and sustained neutralizing antibody and T cell responses in BALB/c mice. In a human DPP4 knock-in (hDPP4-KI) mouse model, it completely protected against lethal challenge with a mouse-adapted MERS-CoV (MERS-CoV-MA). Passive transfer of immune sera to naïve hDPP4-KI mice also provided survival advantages from lethal MERS-CoV-MA challenge. Analysis of sera absorption and isolated monoclonal antibodies from immunized mice demonstrated that the potent and broad neutralizing activity was largely attributed to antibodies targeting the receptor binding domain (RBD) of the S protein. These results show that AdC68-S can induce protective immune responses in mice and represent a promising candidate for further development against MERS-CoV infection in both dromedaries and humans.

## Introduction

The outbreaks of MERS-CoV in Saudi Arabia in 2012 and SARS-CoV in China in 2003 introduced two highly pathogenic coronaviruses into the human population [1,2]. Soon after the initial identification, MERS-CoV epidemic spread to many other countries outside the Arabian Peninsula through infected travellers and most notably in South Korea in 2015 [3]. As of February, 2019, 2374 confirmed cases of MERS and 823 associated deaths were reported with an estimated fatality rate as high as 35% [4]. Like that of SARS infection [5], asymptomatic MERS cases have also been reported [6] suggesting that disease development is likely dependent upon health status and possibly genetics of the infected individual. Up till today, there are still ongoing reports of human MERS-CoV infections in the affected regions. Many are linked to direct contact with dromedaries, which are believed to be a major reservoir host for MERS-CoV and the immediate source of human infection [7,8]. As dromedaries are critical livestock and vital means of transportation in the affected regions, infection and persistence of MERS-CoV in these animals represent a long-term global health threat, highlighting the urgent need for effective prophylactic and therapeutic interventions.

Like that of SARS-CoV, the S protein of MERS-CoV plays a critical role in mediating viral entry and in inducing a protective antibody response in infected individuals and experimental animals [9,10]. The S protein is a typical Type I membrane glycoprotein consisting of a globular S1 domain at the N-terminal region, followed by the membrane-proximal S2 domain and a transmembrane domain [10,11]. Determinants of host range and cellular tropism are located in the receptor-binding domain (RBD) within the S1 domain, while mediators of membrane fusion have been identified within the S2 domain [10–14]. MERS-CoV enters host airway epithelial cells through interaction of RBD with the cellular receptor dipeptidyl peptidase 4 (DPP4) and fusion with either the plasma or endosomal membrane [15]. We and others recently characterized the crystal structure of MERS-CoV RBD bound to the extracellular domain of human DPP4 [16,17]. These studies show that MERS-CoV RBD consists of a core and a receptor binding subdomain. The receptor binding subdomain directly interacts with blades 4 and 5 of the DPP4 propeller but not its intrinsic hydrolase domain [16,17]. This suggests that agents capable of disrupting such binding interaction could serve as candidates to block the entry of MERS-CoV into the target cell. Indeed, both polyclonal and monoclonal antibodies directed against RBD and DPP4 have been shown to inhibit MERS-CoV infection of primary human bronchial epithelial cells and Huh-7 cells [9,15,18]. In particular, we and others have isolated close to twenty neutralizing monoclonal antibodies that target the RBD of the MERS-CoV S protein and interfere with the binding of the cellular receptor DPP4 [19–27]. Crystal structure analyses of these neutralizing antibodies reveal their spatial overlaps and competition for binding with DPP4 [9,28,29]. While neutralizing antibodies remain as a promising option to prevent and treat MERS-CoV infection, the cost associated is relatively high. Vaccine candidate able to induce the type of neutralizing antibodies targeting the RBD would be highly preferred. Therefore, both the S protein and RBD are critical components in various vaccine formulations under investigation aiming to induce the type of neutralizing antibodies mentioned above [30–35]. Reported vaccine candidates directed against the RBD and S protein have been shown to elicit neutralizing activity against MERS-CoV *in vitro* and protective activity in various animal models [35–37]. However, most of these candidates were hampered by limited immunogenicity, and often required multiple rounds of immunization to induce detectable neutralizing antibody or to protect against viral challenge [34,38].

The current study aims to develop vaccine candidate capable of inducing potent and protective immunity against MERS-CoV through single immunization. To this end, we sought to generate a recombinant, rare serotype chimpanzee adenovirus 68 (AdC68) that expresses the RBD-containing full-length MERS-CoV S protein (AdC68-S). Immunogenicity and protective activity of AdC68-S were systematically evaluated against lethal challenge with a mouse-adapted MERS-CoV (MERS-CoV-MA) in our previously developed human DPP4 knock-in (hDPP4-KI) mouse model [39]. Of the chimpanzee adenoviruses, we chose AdC68 as a vector due to its low pre-existing immunity and overall safety profile [40–42]. AdC68 has only 0–2% seropositivity in humans, compared with human adenovirus serotype 5 (HuAd5) which has 75–80% seroprevalence [41]. Recombinant vaccines based on AdC68 and other rare serotype chimpanzee adenoviral vectors, such as ChAd63 and ChAd3, have recently been engineered to express various antigens and some have demonstrated impressive safety and immunogenicity profiles in clinical studies [43–45]. Here, we report that a single intranasal immunization with AdC68-S induces robust and sustained neutralizing antibody and T cell responses in BALB/c mice, superior to commonly used intramuscular vaccinations. In a human DPP4 knock-in (hDPP4-KI) mouse model, the induced immune response completely protected against lethal challenge with a mouse-adapted MERS-CoV (MERS-CoV-MA). Furthermore, passive transfer of immune sera to naïve hDPP4-KI mice also provided survival advantage against lethal MERS-CoV-MA challenge. Through a series of serum absorption and monoclonal antibody isolation from immunized animals, we show that the potent and broad neutralizing activity in immunized animals is largely attributed to antibodies targeting the RBD of the S protein. These results show that AdC68-S can induce desired and favourable antibody responses capable of disrupting interaction between RBD and receptor DPP4. AdC68-S therefore represents a promising candidate for further development against MERS-CoV infection in both dromedaries and humans.

## Materials and methods

*Cells, viruses, and animals*. HEK 293A and HEK 293 T cells represent two different subclones of the parental 293 cell line originated from human embryonic kidney (HEK) cells. HuH-7 cell is a hepatocyte carcinoma cell line originally derived from a liver tumor. All these cell lines were purchased from ATCC and maintained in complete Dulbecco’s modified Eagle’s medium (DMEM, Gibco) supplemented with 10% foetal bovine serum (FBS, Gibco) and incubated at 37°C under 5% CO_2_. The MERS-CoV S protein gene sequence (GenBank number JX869059) was optimized for expression in adenoviral vectors and chemically synthesized (Genscript, China). The mouse-adapted strain of MERS-CoV was generated as previously described and used at the University of Iowa [39]. Six-week old female BALB/c mice were purchased from Beijing Vital River Laboratory Animal Technology Co., Ltd (licensed by Charles River) and housed in specific pathogen free (SPF) mouse facilities. SPF human DPP4 knock-in (hDPP-KI) mice were generated as previously described [39] at the University of Iowa. The hDPP4-KI mice were lightly anesthetized using isoflurane and intranasally infected with a lethal dose (2000 PFU) of MERS-CoV-MA in 50 μL DMEM. All work with MERS-CoV-MA was conducted in Biosafety Level 3 (BSL 3) animal laboratories at the University of Iowa.

*Ethics statement*. All experiments were carried out in strict compliance with the Guide for the Care and Use of Laboratory Animals of the Peoples Republic of China and approved by the Committee on the Ethics of Animal Experiments of Tsinghua University. All animal and neutralization experiments with MERS-CoV-MA were performed in BSL 3 facilities at the University of Iowa Biosafety per the institute for animal care and use committee (IACUC) guidelines. The institutional biosafety committee approved all experiments involving MERS-CoV-MA.

*Construction of recombinant adenoviral vectors*. The MERS-CoV codon-optimized S gene (MERS-S) was cloned into pShuttle-MERS-S and digested with I-CeuI and PI-SceI to release the MERS-S fragment with CMV promoter. The extended fragment containing the MERS-S fragment was separated in agarose gel and inserted into the I-CeuI/PI-SceI sites of pAdC68 to generate pAdC68-MERS-S.

*Rescue, amplification, and purification of recombinant chimpanzee adenovirus*. The type of AdC68 used in our study is a replication deficient chimpanzee adenovirus due to the deletion of the E1 domain in the viral genome. The defective form could only be rescued by the E1 protein expressed and supplied by the HEK 293A cell line. Specifically, HEK 293A cells were seeded on a 6-well plate 1 day before transfection. Cells were cultured overnight to 80–85% confluency at 37 °C and 5% CO_2_ in DMEM with 10% FBS and 1× penicillin–streptomycin solution. pAdC68-MERS-S was linearized with PacI and then transfected into HEK 293A cells using Lipofectamine 2000 according to the manufacturer’s instructions (Life Technologies, Carlsbad, CA). The rescued replication deficient recombinant chimpanzee adenovirus AdC68-S virus was further amplified in HEK 293A cells which were stably integrated with the E1 expression gene. Viruses were purified by a round of CsCl gradient ultracentrifugation followed by desalting with Bio-Gel P-6 DG Media (Bio-Rad, USA). Similarly, the empty AdC68 was generated without any target gene inserted into the vector and purified as described above.

*Detection of MERS-CoV S expression*. HEK 293 T cells infected by AdC68-S or AdC68 virus were used to detect MERS-CoV S expression by Western Blot and by surface staining using flow cytometry. Specifically, HEK 293 T cells were seeded on a 6-well plate one day before transfection, grown to the log phase and infected with either AdC68-S or AdC68 virus at varying doses of 10^8^, 10^9^, and 10^10^ vps/well. Twenty-four hours post-infection, cells were harvested and lysed in 100ul RIPA buffer containing a cocktail of protease inhibitors. Western blots to detect the expression of the MERS-CoV spike protein were run with an anti-MERS-CoV S1 polyclonal antibody (Sino Biological, China). β-Actin immunoblotting was included as a control. Proteins extracted from HEK 293T cells infected with AdC68 viruses were used as a negative control. For cell surface analysis, our previously isolated three monoclonal antibodies (MERS-4, MERS-27, and MERS-GD27) specific for MERS-CoV S were used to stain AdC68-S or AdC68 virus infected cells at 100nM. One hour later, cells were washed and stained with the secondary antibody Anti-human IgG-PE (Santa Cruz Biotechnology, USA) at 1:200. One hour later, cells were washed again and analysed by BD Calibur Facs cytometer (BD, USA). Antibody 17b, specific for human immunodeficiency virus type I (HIV-1), was used as a negative control.

*Immunization and sample collection*. A total of two batches of animals were immunized. In Batch I, mice were randomly distributed into twelve groups (*n* = 5 per group) and vaccinated with varying doses of recombinant vaccine candidates, control viruses, and control PBS via either intramuscular (i.m.) or intranasal (i.n.) route as described in Figure 2(A). Mouse sera were harvested every 2 weeks until the 40th week. In Batch II, mice were randomly distributed into four groups (*n* = 5 per group) and vaccinated with 10^9^vps by i.m. or i.n. route as described in Figure 2(A). Mouse sera were harvested every 2 weeks until the 14th week. Three mice from each group were sacrificed 14 weeks after immunization and their spleens were harvested for cytokines release analysis. The sera were heat-inactivated at 56°C for 30 min before analysed for MERS-CoV S1 specific antibodies.

*Antibody subclass and T cell responses in immunized animals.* MERS-CoV S1 specific IgG, IgG1, IgG2a, and IgA antibody responses were detected by ELISA. Briefly, serially diluted mouse sera were added to 96-well microtiter plates pre-coated with MERS-S1 protein produced in HEK 293 T cells (100 ng/well). Plates were incubated at 37°C for 1 h and washed 3 times with PBST before incubated with either horseradish peroxidase conjugated anti-mouse IgG (1:5000, Promega), IgG1(1:40,000, Abcam), IgG2a(1:40,000, Abcam), or IgA (1:40,000, Abcam) at 37°C for 1 h. Samples were washed 3 times in PBST (Phosphate Buffer Saline-Tween 20). TMB (3′,3′,5′,5′-Tetramethyl Benzidine) was then added to the plates and the reaction was stopped by adding 1M H_2_SO_4_. Absorbance was read at 450 nm using an ELISA plate reader. The ED50 value was calculated based on binding curves drawn in GraphPad (GraphPad Software Inc). To evaluate antigen-specific T-cell responses, 1 × 10^6^ splenocytes per well were stimulated by 1 μM MERS-CoV peptide S291 for 24 h. Secreted TNFα and IL-2 in the culture supernatant were analysed by TNFα/IL-2 ELISA kits (Dakewe, China).

*Epitope mapping through competitive binding between antibodies*. Antibodies 1A8, 1C11, 1D5, and 2C1 were first labelled with biotin. Then, antibodies MERS-4, MERS-27, 17b, MERS-GD27, 1A8, 1C11, 1D5, and 2C1 were serially diluted three-fold before biotin-labelled antibody (50 μl) was added at fixed concentrations. The mixture of antibodies was applied to S1 protein-coated plates, and incubated at 37°C for 1 h. After thorough wash with PBST, 250 ng/ml of streptavidin-HRP (Sigma, USA) were added and incubated at ambient temperature for 30 min before developed with substrate TMB as described above.

*Pseudovirus and live virus neutralization assay*. Neutralizing titres of the immunized mouse sera were determined using a previously reported MERS-CoV pseudovirus and live virus assay [16]. For pseudovirus assay, sera samples were serially diluted 3-fold in 96-well cell culture plates before MERS-CoV pseudovirus was added and incubated at 37°C for 1 h. The number of 1.5 × 10^4^ Huh7 cells were then added to each well and incubated at 37°C for 72 h. The neutralization results were measured in luciferase activity in relative light units (Vigorous, China). For live virus assay, sera samples were collected as described above and serially diluted in 48-well cell culture plates. MERS-CoV/MERS-CoV-MA was added to each well and incubated at 37° for 1 h before added to 12-well plates pre-coated with Vero E81 cells and incubated at 37°C for 3 days. The neutralization titres were determined by crystal violet staining.

*Lung virus titres*. To determine lung virus titres, hDPP4-KI mice infected with MERS-CoV-MA were euthanized at 4-days post-challenge. Lungs were removed after transcardial perfusion with PBS and homogenized in PBS. Viral titres were determined on Vero E81 cells and represented as plaque-forming units (PFU)/lung. Cells were fixed with 10% formaldehyde and stained with crystal violet at 3 d.p.i.

*Sequence analysis and statistics*. Sequence alignments were performed with BioEdit (BioEdit Software Inc). Phylogenetic analysis was generated using program MEGA (MEGA Software Inc). Half-maximal inhibitory dilutions (ID50) and half-maximal effective dilutions (ED50) were calculated for each serum using GraphPad Prism 5 software (GraphPad Software Inc). IC50 for monoclonal antibodies were estimated by GraphPad. Data were expressed by means or means ± standard deviations and significance in their differences were analysed using Student’s t-test. Statistical significance for survival studies was calculated using the log-rank (Mantel–Cox) test with 95% confidence intervals (CI). Results are graphs with mean ± SEM.

## Results

### Construction and characterization of a recombinant chimpanzee adenovirus vaccine expressing MERS-CoV spike glycoprotein

**Figure 1.**
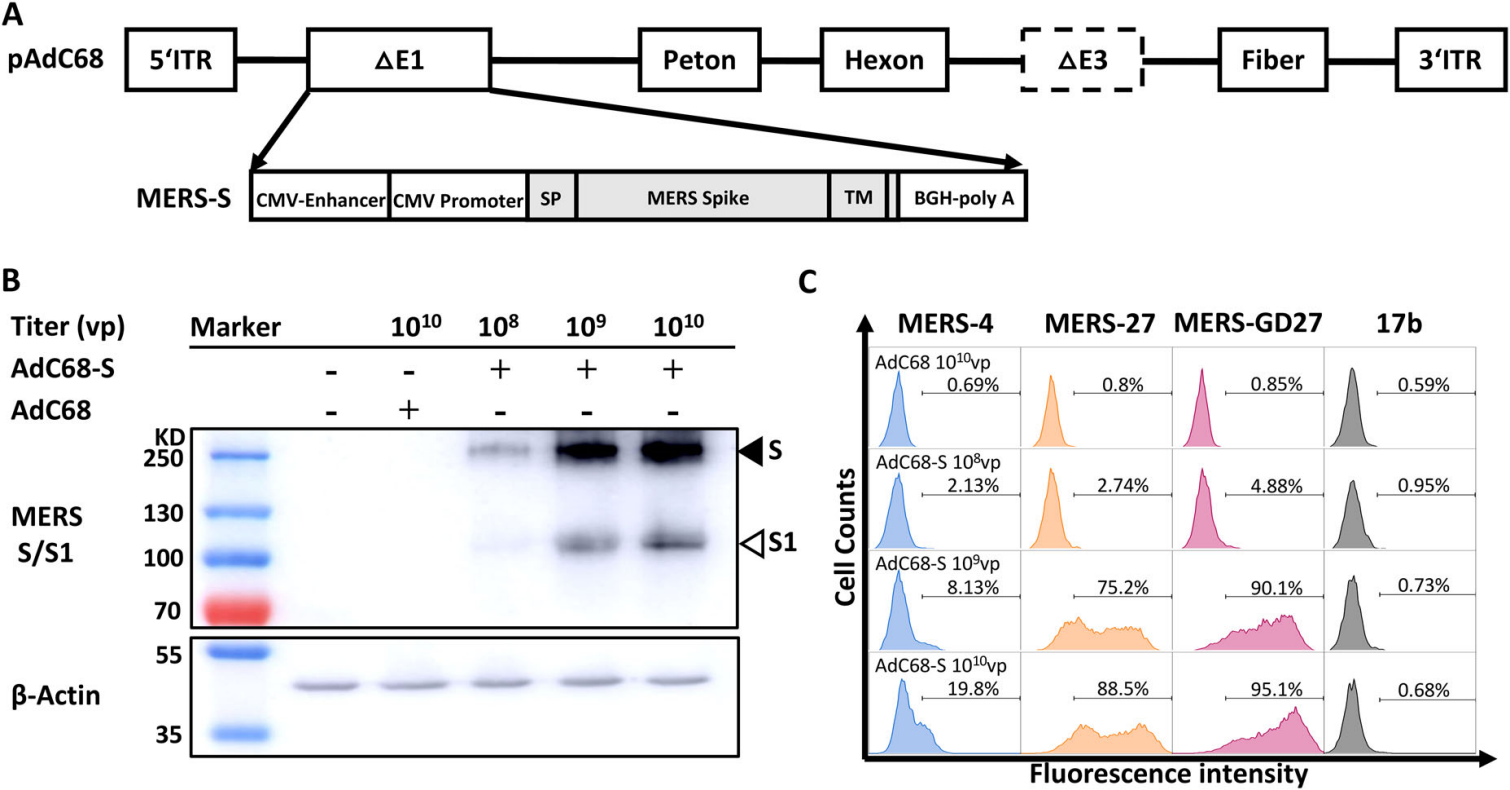
Generation and evaluation of recombinant AdC68 expressing full-length MERS-CoV spike protein. (A) Schematic representation of the recombinant AdC68 expressing the full-length MERS-CoV spike gene (AdC68-S) (Genbank accession number: JX869059). Spike gene was inserted into the E1 region of AdC68 under the control of the CMV promoter and terminated by bovine growth hormone (BGH) polyadenylation signal sequence. (B) Western blot analysis of MERS-CoV S protein expression in 293 T cells after infection with AdC68-S (10^8^, 10^9^ and 10^10^ vp). MERS-S protein in cell lysates was probed by Rabbit anti-MERS-S1 polyclonal antibody (Sino biological). The solid arrow points to the band of S protein while the hollow arrow points to the band of S1 protein cleaved from S by the protease. (C) Cell surface expression of MERS-CoV S protein analysed by MERS-CoV-specific antibodies MERS-4, MERS-27, MERS-GD27. Cell lysates or cells infected by empty AdC68 (10^10^vp) were used as negative controls. 17b, an antibody against HIV-1, was used as a negative control antibody.

We constructed a recombinant chimpanzee adenovirus vaccine by cloning the full-length MERS-CoV spike gene into the E1 deletion region of the AdC68 genome (AdC68-S). The spike gene was codon-optimized based on a human MERS-CoV isolate (JX869059) under the control of the CMV promoter (Figure 1(A)). The recombinant AdC68-S virus was produced in HEK 293A cells, purified by CsCl gradient ultracentrifugation, and quantified as viral particles (vp) by measuring the wavelength absorbance at 260 nm. To assess spike protein expression, we performed Western blot analysis of HEK 293 T cells lysates infected with AdC68-S using a rabbit polyclonal anti-MERS S1 antibody. As shown in Figure 1(B), we detected dose-dependent expression of the spike protein in two prominent forms. One had a high molecular weight (∼200,000–210,000 MW) representing the full-length spike glycoprotein and the other had a low molecular weight (∼1,20,000 MW) corresponding to the S1 subunit of cleaved spike glycoprotein. Cell surface staining was also carried out to confirm the S protein expression on the AdC68-S infected HEK 293 T cells, using antibodies specific for the RBD of MERS-CoV previously isolated by our group (MERS-4, MERS-27, and MERS-GD27) [21,23] (Figure 1(C)). These results demonstrate successful and robust expression of the MERS-CoV S protein on the surface of and within AdC68-S infected cells.

### A single immunization with AdC68-S induces potent humoral and cellular immune response against MERS-CoV

**Figure 2.**
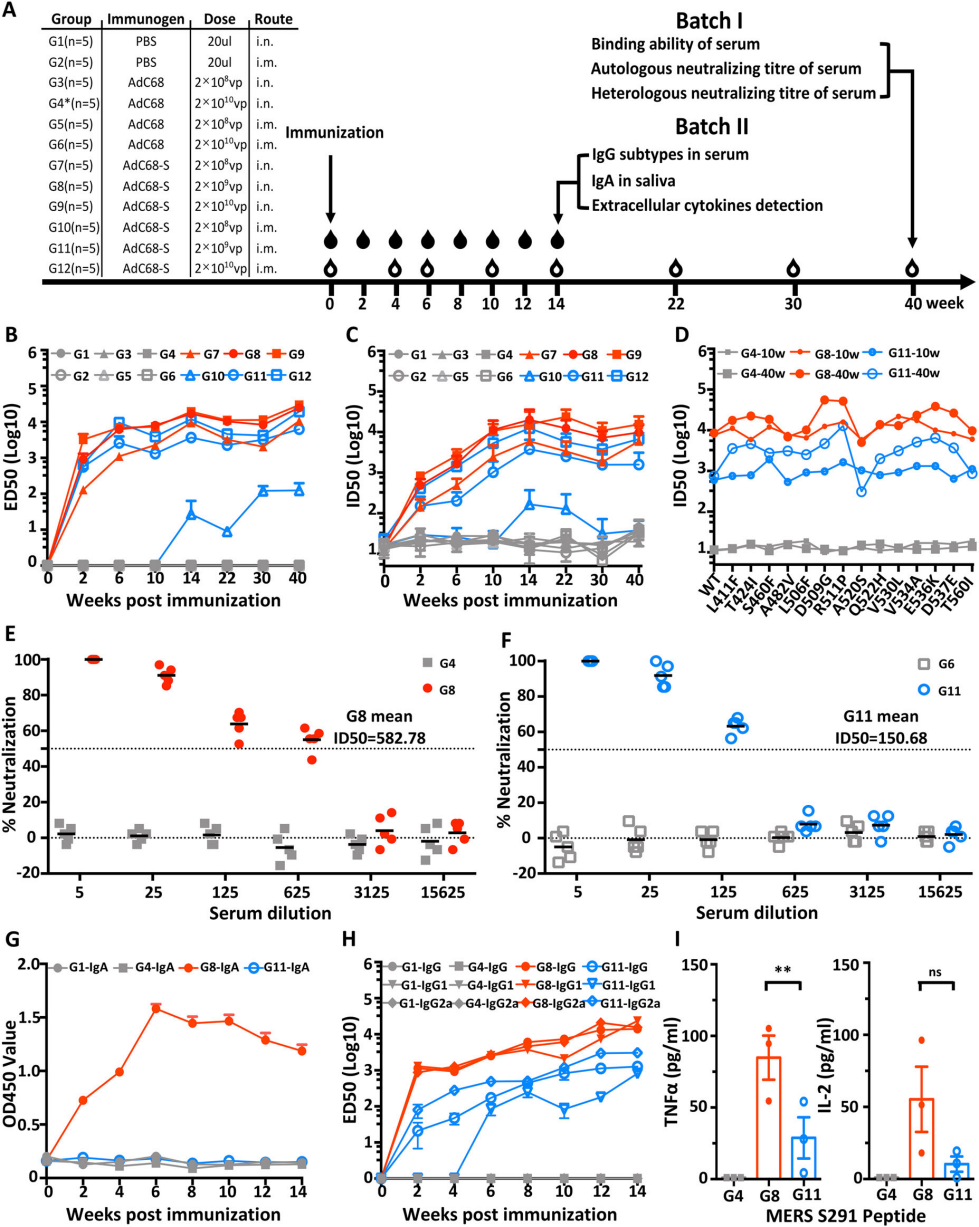
Intranasal immunization with AdC68-S induces a robust antibody and T cell response in BALB/c mice. (A) Timeline for vaccination and characterization of virologic and immunologic responses in two batches of animals. In batch I, a total of 12 groups of mice were immunized and monitored for serum binding and neutralizing activities. Group 1–6 (G1-G6) were negative controls whereas group 7–12 (G7–G12) were vaccinated with a single and varying dose of AdC68-S. In batch II, G8 and G11 mice were immunized along with G1 and G4* controls mice. These mice were examined for serum IgG subtypes, saliva IgA, and cytokine release up to 14-weeks post-immunization. The specific dose and route of immunization are indicated. i.n.: intranasal. i.m.: intramuscular. The open and solid drops indicate the blood collection for animals in the batch I and II, respectively. The temporal changes in serum binding activity to MERS-S1 (B) and neutralizing activity against autologous (C) and heterologous (D) MERS-CoV variants up to 40-weeks post-immunization are shown. Live virus neutralization by immune sera from G4, G8 (E) and G6, G11 (F) at 40-weeks post-immunization. The mean ID50 for G8 and G11 immune sera are indicated. The temporal changes in IgA in saliva (G), serum IgG subtypes (H), and cytokine release (I) in G8 and G11 animals were studied for up to 14-weeks post-immunization. Red symbols represent sera from i.n. vaccination groups and blue colour indicates sera from i.m. immunized animals. The grey colour is indicative of the control groups. ED50 means dilutions of serum at which half of the binding to antigen was identified. ID50 means dilutions of serum at which half of the viruses are neutralized. G4* indicates 10^10^vp used in batch I and 10^9^ in batch II.

Next, we studied the impact of dose and route of vaccination on the immunogenicity of AdC68-S in BALB/c mice. The overall scheme for group design, immunization, and immunological characterization is outlined in Figure 2(A). The experiments were conducted in two consecutive batches. In the first, all 12 groups of mice (G1-G12) were followed up to 40 weeks. The second was guided by the results obtained from the first batch and focused on G8 and G11 up to 14 weeks after immunization. For animals in Batch I, a single immunization of AdC68-S in a high (2 × 10^10^ vp), medium (2 × 10^9^ vp), or low (2 × 10^8^ vp) dose was administered through either an intramuscular (i.m.) or intranasal (i.n.) route. Sequential sera samples were collected at weeks 0, 4, 6, 10, 14, 22, 30, and 40 post-vaccination and analysed for binding and neutralizing activity against pseudotyped and live MERS-CoV. As shown in Figure 2(B), all immunized groups with AdC68-S except G10 developed high levels of S1-specific binding antibodies (ED_50_) as early as week-two post-immunization. The antibody response continued to increase and persisted up to 40-weeks post-immunization (w.p.i.) (Figure 2(B) and Fig. S1A). These high binding levels coincided with potent and broad neutralizing activities against MERS-CoV pseudovirus carrying wildtype and naturally occurring mutant envelopes (Figure 2(C,D)). The ID_50_ of immune sera against the wildtype pseudoviruses reached as high as 10^4^ dilutions around 10 weeks and persisted to similar or increased to higher levels at 40 w.p.i. (Figure 2(C) and Fig. S1A). In addition, 10 and 40-weeks immune sera from G8 and G11 demonstrated potent and broad neutralizing activities against 14 major mutant pseudoviruses derived from 175 naturally occurring MERS-CoV spike sequences. The average ID50 for 10-weeks immune sera was 1018.6 for i.m. and 10,751.3 for i.n. and that for 40 weeks was 3767.7 and 21,442.1 respectively (Figure 2(D) and Fig. S1B). Importantly, the same immune sera also demonstrated potent neutralizing activities against live MERS-CoV. Consistent with results from the pseudovirus assay, the ID50 of 40-weeks immune sera from G8 and G11 reached average of 582.78 and 150.68 respectively while that from the vector control groups G4 and G6 had no detectable neutralizing activity (Figure 2(E,F)). Furthermore, we found that intranasal immunization induced a stronger antibody response compared with the intramuscular route in all dose groups (Fig. S1A and S1B). For example, at 40 w.p.i., the average ID50 for low-dose groups is 5929.8 for i.n. (G7) and <45 for i.m. (G10), 9785.6 for i.n. (G8) and 1583.3 for i.m. (G11) for medium-dose group, and 15,452 for i.n. (G9) and 6739.2 for i.m. (G12) for high-dose group, respectively. For practical and cost-effective reasons, we selected the medium dose (G8 and G11) for follow-up characterization in Batch II.

In Batch II, BALB/c mice were immunized only with medium-dose 2 × 10^9^ vp of blank AdC68-S, 2 × 10^9^ vp of AdC68, and PBS via the i.n. (G8, G4*, and G1) or i.m. (G11) route. Sequential sera and saliva samples were collected at weeks 0, 2, 4, 6, 8, 10, 12, and 14 post-vaccination and analysed for binding and neutralizing activity against pseudotyped MERS-CoV. As shown in Figure 2(G), only animals immunized with AdC68-S through the i.n. (G8) but not the i.m. route (G11) had detectable IgA responses in saliva. This peaked about 6 w.p.i. and gradually declined over the ensuing period. The same animals in G8 also displayed higher levels of sera IgG1, IgG2a, and total IgG levels compared to those in G11 (Figure 2(H)). As early as 2 w.p.i, IgG1, IgG2a, and total IgG were detectable and continued to rise up to 14 w.p.i.. In comparison, animals in G11 either sustained lower response or had a significantly delayed response, particularly for IgG1 at 14 w.p.i. (Figure 2(F) and Fig. S1C). For analysing MERS-CoV specific T cell responses, we measured levels of secreted TNFα and IL-2 from splenocytes after stimulation with MERS-S291 peptide [37]. At 14 w.p.i., animals in G8 produced significantly higher levels of TNFα and IL-2 compared to those in G11 (Figure 2(G)). These results indicate that a single intranasal immunization with AdC68-S induces a superior antibody and cellular immune response than an equivalent intramuscular immunization. We therefore selected the medium-dose of AdC68-S (2 × 10^9^ vp) and intranasal immunization for subsequent evaluation of protection against live MERS-CoV challenge in a mouse model.

### A single intranasal immunization with AdC68-S protects hDPP4 knock-in mice from the lethal MERS-CoV challenge

**Figure 3:**
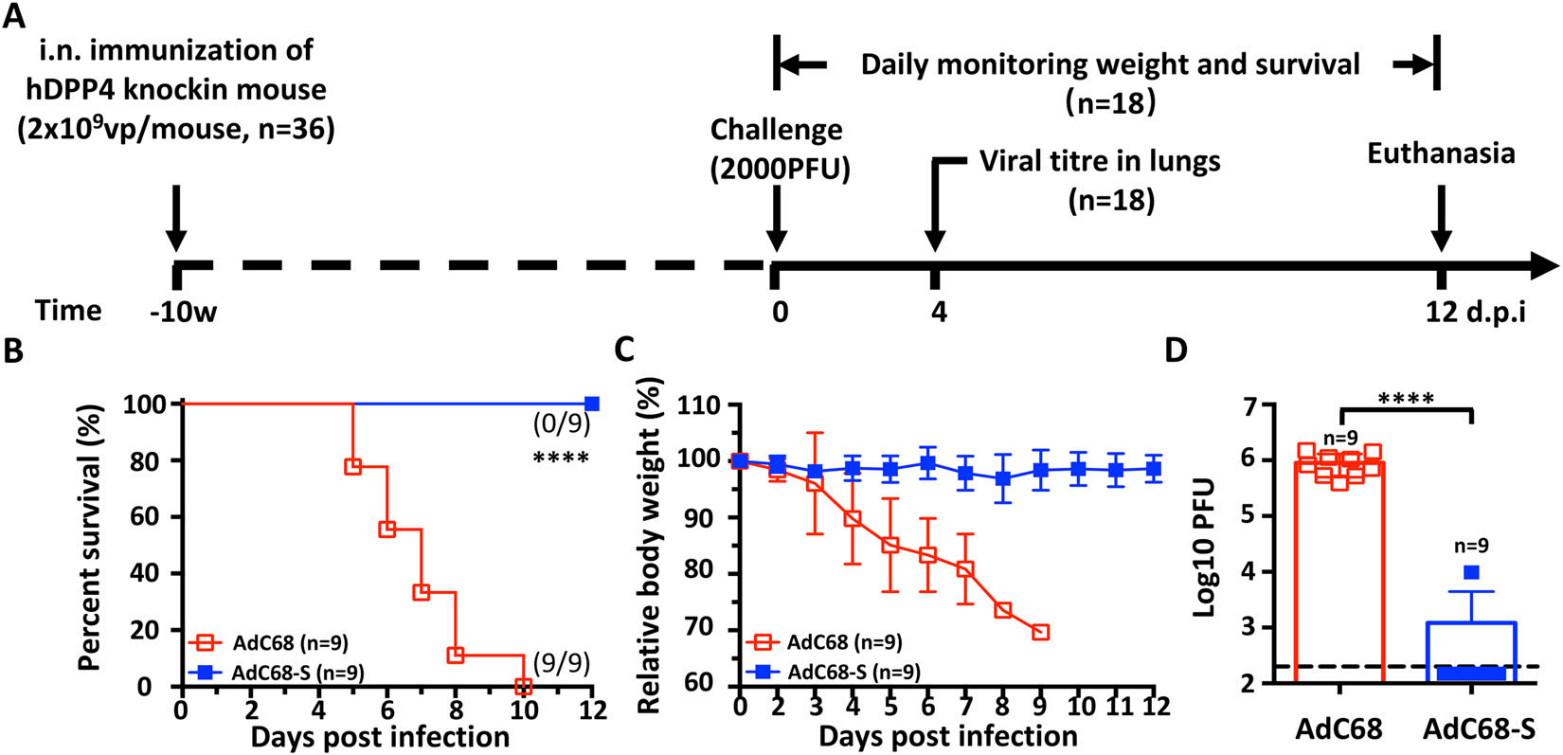
Intranasal immunization with AdC68-S provides complete protection against lethal MERS-CoV challenge in human DPP4 knock-in mice. (A) Timeline for immunization, challenge and evaluation of protective efficacy. Human DPP4 knock-in (KI) mice were immunized with either 2 × 10^9^ vp AdC68-S or empty AdC68 via i.n. route. Ten weeks later, the same set of animals were challenged intranasally with 2000 PFU of mouse-adapted MERS-CoV strain MERS-CoV-MA and monitored daily for (B) survival and (C) weight loss. On day-4 post-infection, lung virus titres (D) were examined. Data are shown as mean ± SEM. *p*-values were analysed with Student’s t-test (****P < 0.0001). The dashed line in (D) indicates the limit of detection.

We next evaluated the protective efficacy of AdC68-S vaccination using a newly developed hDPP4-KI mouse model of fatal MERS-CoV infection (Figure 3(A)) (n = 36) [39]. Specifically, a total of 18 mice received AdC68-S vaccine via intranasal route while the other 18 received blank AdC68 vector as a negative control. Ten w.p.i., all animals were challenged intranasally with a lethal dose of 2000 plaque-forming units (PFU) of mouse-adapted MERS-CoV-MA. Half of the animals (9 out of 18) in each AdC68-S and AdC68 groups were monitored daily for survival and body weight changes throughout a 12-day follow-up while the other half (9 out of 18) were sacrificed for viral load measurement in lungs on day 4 post-challenge (Figure 3(A)). In the 12 d following-up group, all 9 animals receiving AdC68-S vaccine survived challenge and demonstrated relative stable body weight (Figure 3(B,C)). In contrast, negative control animals began losing weight significantly starting on 3 d.p.i. and succumbed to MERS-CoV-MA infection before 10 d.p.i. (Figure 3(B,C)). In the sacrifice group on 4 d.p.i., 8 out of the 9 AdC68-S immunized animals had no detectable viral loads, while control animals had on average as high as 10^6^ PFU of virus in their lungs (Figure 3(D)). Together, these results indicate that a single intranasal immunization with AdC68-S can significantly reduce viral replication and provide complete protection from a lethal MERS-CoV-MA challenge.

### Passive immunization with immune sera protects hDPP4-KI mice from a lethal MERS-CoV challenge

**Figure 4.**
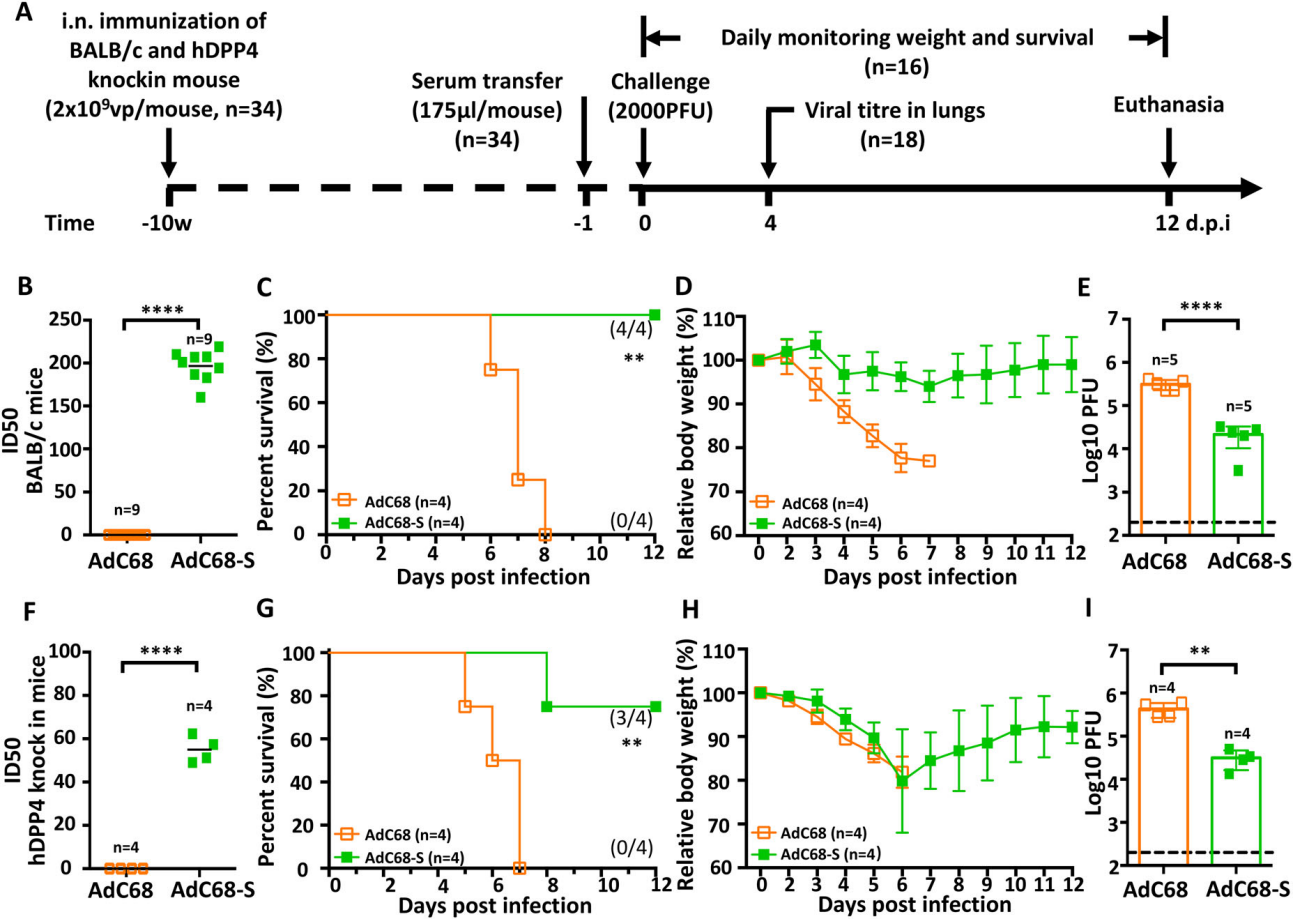
Protective efficacy of passive immunization with AdC68-S immune sera. (A) Timeline of immunization, serum transfer, challenge, and monitoring for various biological and clinical outcomes. BALB/c and human DPP4 KI mice were immunized with either 2 × 10^9^ vp AdC68-S or empty vector AdC68 via i.n. route. 175 µl of immune sera collected from these BALB/c and hDPP4-KI mice were transferred to hDPP4-KI mice via intraperitoneal route one day before lethal 2000 PFU MERS-CoV-MA infection. (B) Neutralizing activity of immune sera from immunized BALB/c (B) and hDPP4-KI (F) mice. (C-E) Survival (C), weight loss (D) and lung viral titres (E) in hDPP4-KI mice receiving sera from immunized BALB/c mice. (G-I) Survival (G), weight loss (H) and lung viral titres (I) in hDPP4-KI mice receiving sera from immunized hDPP4-KI mice. Data are mean ± SEM. *p*-values were analysed with Student’s t-test (**P < 0.01; ****P < 0.0001). The dashed line in (E) (I) indicates the limit of detection.

To evaluate the antibody contribution to the observed protection, we transferred immune sera from either BALB/c or hDPP4-KI mice immunized with AdC68-S to naïve, non-immunized hDPP4-KI mice as illustrated in Figure 4(A) (n = 34). The neutralizing titre against mouse-adapted MERS-CoV-MA was about 4-fold higher in immunized BALB/c than in hDPP4-KI mice (Figure 4(B,F)), perhaps due to intrinsic differences between the two animal species or the effect of DPP4 knock-in. A total of 175 µl of immune sera from each and every immunized mouse at 10 w.p.i. was transferred individually into each of hDPP4-KI mice through intraperitoneal (i.p.) injection one day before challenge with a lethal dose (2000 PFU) of MERS-CoV-MA. Sixteen out of 34 animals receiving either BALB/c or hDPP4-KI immune sera were monitored daily for survival and body weight changes throughout a 12-day follow-up while the rest 18 were sacrificed for viral load measurement in lungs on day 4 post-challenge (Figure 4(A)). Consistent with neutralizing activity, immune sera from BALB/c mice provided 100% (4/4) protection, while that from hDPP4-KI mice resulted in 75% (3/4 mice) survival (Figure 4(C,G)). There were no significant declines in body weight in animals that received immune sera from BALB/c mice (Figure 4(D)). However, animals that received hDPP4-KI mouse immune sera lost approximately 20% of their body weight during the first 6 days after infection, then gradually recovered in the ensuring period (Figure 4(H)). Control hDPP4-KI mice that received sera samples from empty vector AdC68 immunized-animals lost weight and then succumbed to MERS-CoV-MA infection by 8 d.p.i. (Figure 4(C–D,G–H)). In the sacrifice group on 4 d.p.i, all mice that passively received immune sera from AdC68-S immunized mice had significantly lower virus titres in lungs than those from empty vector AdC68 immunized mice sera (Figure 4(E,I)). Together, these findings confirm that the antibody response induced by AdC68-S plays a seminal role in protecting hDPP4-KI mice from lethal challenge with MERS-CoV-MA.

### Single intranasal immunization with AdC68-S elicits potent and broad RBD-specific neutralizing antibodies

**Figure 5.**
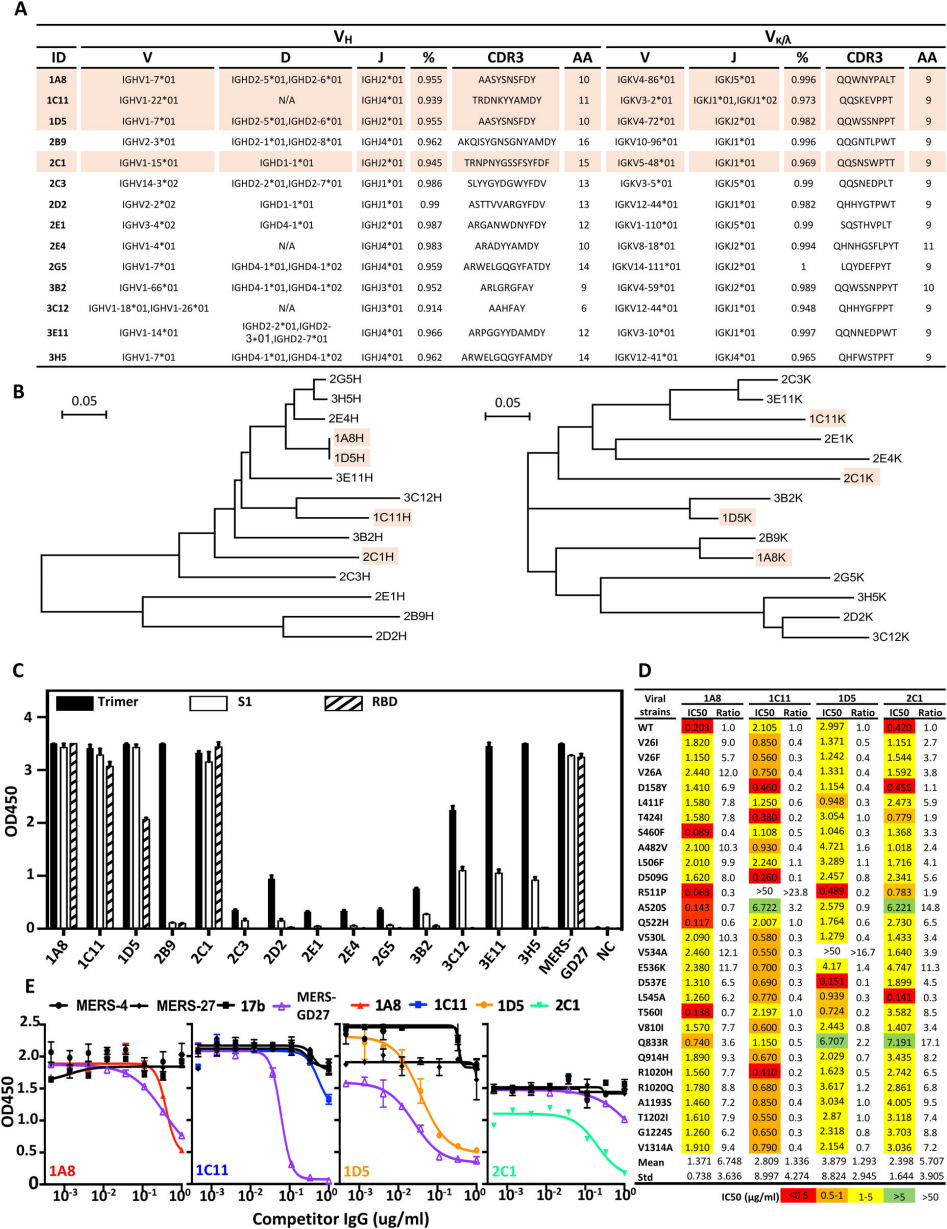
Characterization of AdC68-S-elicited monoclonal antibodies. (A) Summary of isolated 14 mouse mAbs on their family designations and degree of similarity compared to their germline sequences, together with their sequences of complementarity-determining region 3 (CDR3) for both VH and VL. (B) Unrooted neighbor-joining tree depicting the relationship of isolated mAbs, left panel for the heavy chain and right panel for the light chain variable region. The branch length is drawn to scale so that the relatedness between different amino acid sequences can be readily assessed. Individual sequences are named at the tip of the branches. Binding (C) and neutralizing (D) activities of 14 mAbs measured by ELISA and pseudovirus bearing naturally occurring MERS-CoV mutant strains. (E) Epitope specificity analysed by competitive ELISA. HIV-1-specific mAb 17b was used as a negative control whereas previously isolated MERS-CoV-specific human mAb MERS-4, MERS-27 and MERS-GD27 as positive controls. Data are presented as mean ± SEM. The four antibodies with potent neutralizing activities are colored in light orange in (A) and (B).

To further characterize the antibody response induced by AdC68-S, we isolated a total of 14 monoclonal antibodies (mAbs) from antigen-specific single splenocytes of immunized BALB/c mice using MERS-S trimer as a bait. These antibodies are rather divergent in the V_H_ (10V_H_1, 2V_H_2, 1V_H_3, 1V_H_) and V_L_ (1Vκ1, 3Vκ3, 3Vκ4, 1Vκ5, 1Vκ8, 1Vκ10, 3Vκ12, 1Vκ14) gene families (Figure 5(A)) and are distributed widely without apparent clustering on the phylogenetic tree (Figure 5(B)). Both heavy and light variable regions share high degree of similarity with their germline sequences. To study the function of these isolated antibodies, we constructed the full-length antibody on the backbone of mouse IgG1 antibody, produced in HEK 293 T cells, purified and quantified. Their binding activity to the recombinant S trimer, S1, and RBD protein of MERS-CoV was then measured by ELISA. As shown in Figure 5(C), all of the antibodies were reactive to MERS-S trimer, but with significantly different activities. Eight clones (1A8, 1C11, 1D5, 2B9, 2C1, 3C12, 3E11, and 3H5) demonstrated exceedingly high binding activities, while activities of the remainder were relatively low. Among the eight clones, seven (1A8, 1C11, 1D5, 2C1, 3C12, 3E11, 3H5) were reactive to S1 protein and four (1A8, 1C11, 1D5, 2C1) to the RBD (Figure 5(C)). The control mAb MERS-GD27 demonstrated expected specificity, as previously reported by us [23]. However, only the four antibodies reactive to RBD (1A8, 1C11, 1D5, 2C1) demonstrated potent and broad neutralizing activities against both wild type and the panel of major mutant pseudoviruses derived from 175 naturally occurring MERS-CoV spike sequences (Figure 5(D)). The most potent and broad neutralizing activities were found for 1A8 and 2C1 against wild type and mutant strains with mean IC50s 1.371μg/ml and 2.398μg/ml, respectively. On the other hand, 1C11 and 1D5 showed modest neutralizing activity with mean IC50s of 2.809μg/ml and 3.879μg/ml, respectively, and failed to neutralize strains bearing either the R511P or V534A mutation. To map the epitopes of these four mAbs, we conducted competition experiments against antibodies with known epitope specificity, such as MERS-4, MERS-27, and MERS-GD27 published earlier [21,23]. All four neutralizing mAbs competed with MERS-GD27 in variable degrees, but failed to compete with MERS-4 and MERS-27 (Figure 5(E)). This suggests that they approach RBD in a manner similar to MERS-GD27. Also, we conducted sera absorption experiments with RBD and compared neutralization activity before and afterward. After adsorption by RBD, the immunized sera from G8 at 2 w.p.i. lost from 44.7% to 98.2% and at 10 w.p.i. lost from 33.5% to 82.8% of their neutralizing ability against MERS-CoV pseudovirus. In the control group, that sera were adsorbed by a protein of HIV-1 showed no changes in the neutralizing ability (Fig. S2). Together, these findings demonstrate that the observed protection conferred by the sera of immunized animals was largely attributed to antibodies targeting RBD, and highlight the critical role of RBD-directed antibody response in protection.

## Discussion

MERS-CoV continues to infect humans and cause morbidity and mortality with no sign of abating. One contributing factor is the high prevalence of MERS-CoV infection among dromedaries [8,46,47], which are believed to be major reservoirs for the virus and the immediate source of human infection [48]. The persistence of MERS-CoV in these animals poses a severe, long-term threat to global health and highlights the urgent need for prophylactic and therapeutic interventions. In this study, we constructed a recombinant, rare serotype chimpanzee adenovirus 68 (AdC68) that expresses the full-length MERS-CoV S protein (AdC68-S) as a potential vaccine candidate against MERS-CoV infection. We show that single intranasal immunization with AdC68-S induces robust and sustained neutralizing antibody and T cell responses in BALB/c mice. Induced immunity is able to provide complete protection against lethal challenge with MERS-CoV-MA in the hDPP4-KI mouse model. Furthermore, passive transfer of immune sera to naïve hDPP4-KI mice also provides substantial survival advantage against lethal MERS-CoV-MA challenge. Sera absorption analysis and isolation of monoclonal antibodies from immunized mice demonstrate that the observed potent and broad neutralizing activity can be attributed to the antibodies targeting to the RBD of the S protein. These results show that AdC68-S can induce protective immune responses in mice and represent a promising candidate for further development against MERS-CoV infection in both dromedaries and humans.

There are two unique aspects of our study can be highlighted here. The first is related to the AdC68 vector, which has preferred profiles of low pre-existing immunity and overall safety in animals and humans [40–42]. This is clearly advantageous over many human adenoviral vectors that are widely used in vaccine development, such as human adenovirus 5 (HuAd5). Compared to HuAd5, which has 75–80% seroprevalence in humans, AdC68 has only 0–2% seropositivity [41]. Recently, AdC68 has been engineered into vaccine candidates for a wide range of pathogens such as hepatitis C virus [44,49], influenza A virus [45], Ebola virus [50,51], HIV-1 [49,52] and RSV [53], and promising preclinical results have brought AdC68 closer to clinical application. Other serotype chimpanzee adenovirus-based vaccines have demonstrated similar favourable profiles of low pre-existing immunity and some have advanced to clinical studies [42]. Notably, a ChAdOx1 vaccine expressing the full-length of MERS-CoV S protein has already entered phase I human trials [54,55]. A ChAd63 ME-TRAP vaccine against *Plasmodium falciparum* proved to be safe and highly immunogenic in humans [43,56]. ChAd3 encoding the Ebola Zaire glycoprotein (ChAd3-EBO-Z) is safe and induces neutralizing antibodies and T cell responses against the Ebola virus in humans [50]. Furthermore, both PanAd3-RSV, which encodes the RSV proteins F, N, and M2-1, and ChAd3-NSmut, which encodes the hepatitis C virus proteins NS3, NS4, NS5A, and NS5B, elicited strong immune responses in clinical trial participants [44]. These results demonstrate that chimpanzee adenoviral vectors have impressive safety, tolerability, and immunogenicity profiles in humans and highlight their promise for vaccine development [40,41].

The second unique aspect of this study is the exceptional and protective immunogenicity of AdC68-S in mice. Our results show that a single intranasal immunization with AdC68-S induces robust and sustained systemic and mucosal immune responses in BALB/c mice after 40-weeks of immunization. In a hDPP4-KI mouse model, it provided complete protection against lethal challenge with MERS-CoV-MA 10-weeks after initial immunization. Such high and persistent levels of antibody is in great contrast to those induced by other candidate vaccines for which immunogenicity is short-lived and multiple rounds of immunization are required to induce detectable levels of neutralizing antibody or to confer protection against viral challenge [34,38]. Although the exact mechanism behind the long-lasting and sustained immune responses is currently unknown, it is possible that the broad tropism for host cells of AdC68 [57] and membrane-anchored instead of soluble Spike protein expressed by the recombinant AdC68-S had played critical roles. Importantly, intranasal immunization with AdC68-S appears to be superior to intramuscular immunization in inducing mucosal immune responses, although the underlying mechanism remains unclear [58]. This is particularly relevant to MERS-CoV infection as viral transmission occurs largely through the respiratory tract [59,60]. Furthermore, ease of intranasal immunization also offers practical convenience over methods requiring multiple prime and boost injections, and thereby improves population coverage. In the previous study utilizing chimpanzee adenoviruses intramuscular immunizations appeared to elicit better immune responses/protection than intranasal inoculations [54]. This difference may be caused by the different serotype of the adenovirus vector, actual antigen and animal model used. Both neutralizing antibody and T cell responses appeared to play important and synergistic roles in protective immunity. Neutralizing antibodies likely to directly inhibit MERS-CoV entry and/or function through Fc receptor mediated functions such as antibody-dependent cellular cytotoxicity (ADCC) largely through nature killers (NK) cells and antibody-dependent phagocytosis (ADPC) largely through neutrophils. T cells, on the other hand, would be expected to recognize and kill MERS-CoV infected cell through recognition of MHC-I presented peptide such as S291 tested in the current study. However, passive transfer of immune sera to naïve hDPP4-KI mice provided substantial protection against lethal MERS-CoV-MA challenge. Thus, it is reasonable to hypothesize that antibodies and their mediated effector functions may be sufficient in protection. In particular, analysis of sera absorption and isolation of monoclonal antibodies from immunized mice indicate that the protective response can be largely attributed to antibodies targeting to the RBD of the S protein. This coincides with previous reports in which vaccine candidates based on RBD alone can induce protective antibody responses against MERS-CoV infection [35,61–65]. It is also consistent with earlier findings that neutralizing monoclonal antibodies targeting RBD are sufficient to provide protection [20,22,26,27,35]. Collectively, our results demonstrate that AdC68-S has exceptional capacity to induce protective immune response in mice and notable ease of delivery. It represents a promising candidate for further development against MERS-CoV infection in both dromedaries and humans.

## Supplementary Material

Supplemental Material
