# Supplementary figures and images for: Single intranasal immunization with chimpanzee adenovirus-based vaccine induces sustained and protective immunity against MERS-CoV infection
Source: Emerg Microbes Infect. 2019 May 25;8(1):760–72. doi: 10.1080/22221751.2019.1620083 (PMC6542157; doi:10.1080/22221751.2019.1620083)

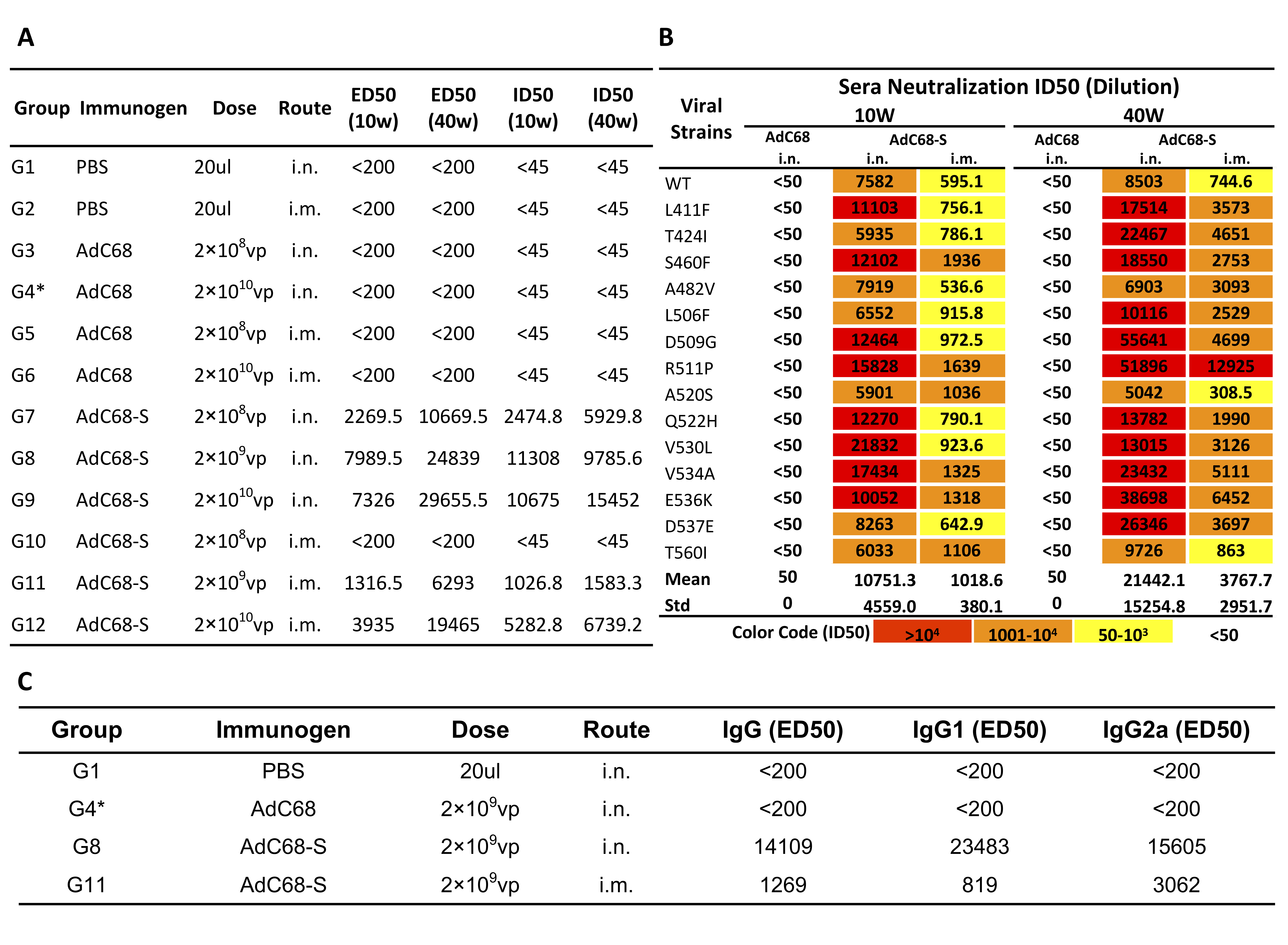

Supplement: Supplemental Material [file TEMI_A_1620083_SM0563.zip › FigureS1.tiff]

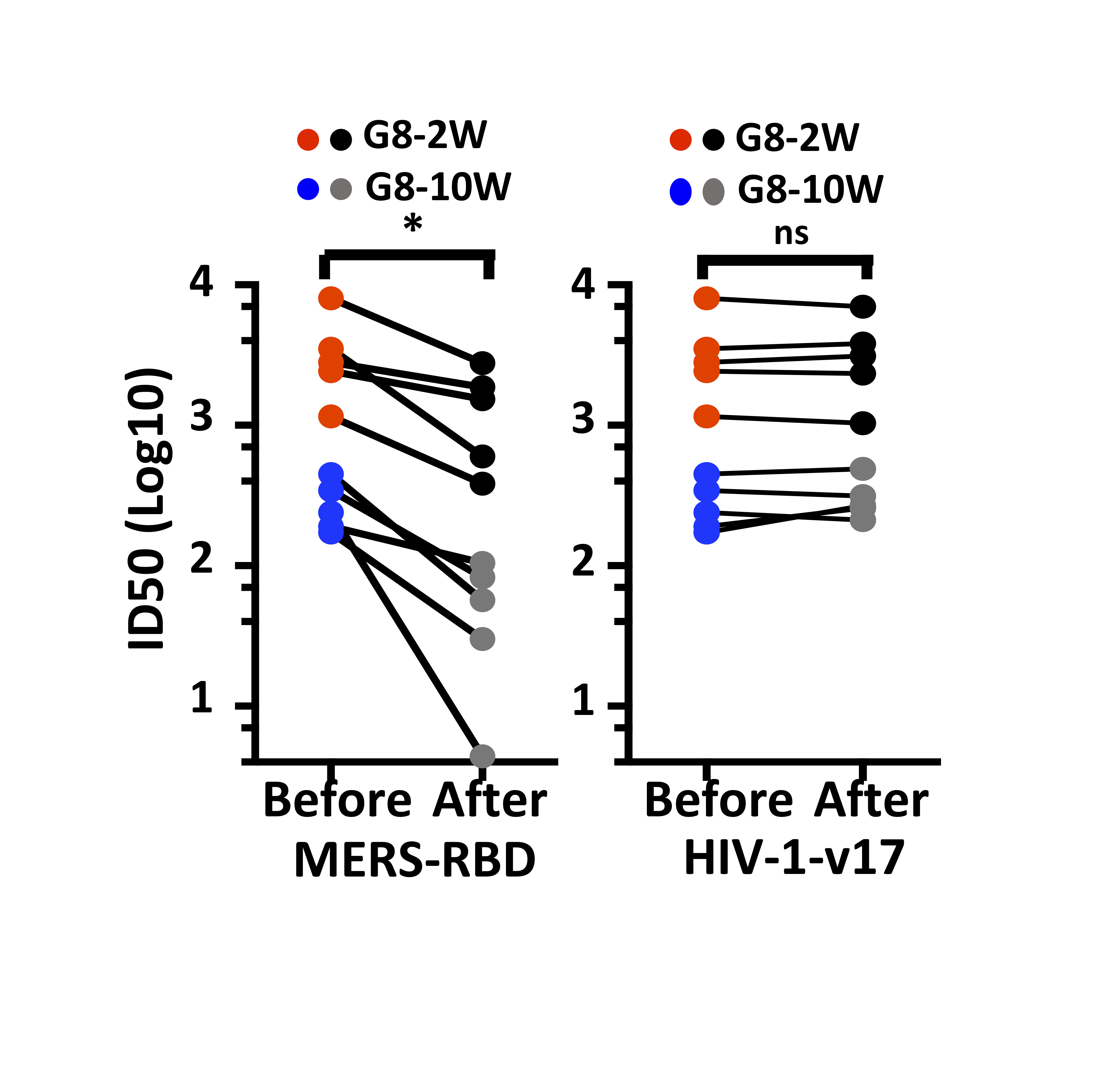

Supplement: Supplemental Material [file TEMI_A_1620083_SM0563.zip › FigureS2.tiff]
